# Anthracyclines disaggregate and restore mutant p63 function: a potential therapeutic approach for AEC syndrome

**DOI:** 10.1038/s41420-025-02307-0

**Published:** 2025-01-25

**Authors:** Fabiana Boncimino, Ludovica D’Auria, Kristina Todorova, Sabina Y. van der Zanden, Jacques Neefjes, Anna Mandinova, Caterina Missero, Stefano Sol

**Affiliations:** 1 Cutaneous Biology Research Center, Massachusetts General Hospital and Harvard Medical School, Charlestown, MA 02129 USA; 2 CEINGE Biotecnologie Avanzate Franco Salvatore, 80145 Naples, Italy; 3 Department of Biology, University of Naples Federico II, 80126 Naples, Italy; 4 Department of Cell and Chemical Biology, ONCODE Institute, Leiden University Medical Center, 2333 ZC Leiden, The Netherlands; 5 Broad Institute of Harvard and MIT, 7 Cambridge Center, Cambridge, MA 02142 USA; 6 Harvard Stem Cell Institute, 7 Divinity Avenue, Cambridge, MA 02138 USA

**Keywords:** High-throughput screening, Protein aggregation

## Abstract

Ankyloblepharon-Ectodermal Defects-Cleft Lip/Palate (AEC) syndrome is a rare genetic disorder caused by mutations in the TP63 gene, which encodes a transcription factor essential for epidermal gene expression. A key feature of AEC syndrome is chronic skin erosion, for which no effective treatment currently exists. Our previous studies demonstrated that mutations associated with AEC syndrome lead to p63 protein misfolding and aggregation, exerting a dominant-negative effect. By performing a high-throughput screening of epigenetic and FDA-approved compounds in a co-transfection model of wild-type and mutant p63, we found that two compounds, Doxorubicin and Epirubicin, alleviate protein aggregation and restore p63 transactivation function. Moreover, treatment with these compounds reduced protein aggregation and restored the expression of keratinocyte-specific p63 target genes in primary keratinocytes derived from a conditional ΔNp63αL514F knock-in AEC mouse model, which mimics the ectodermal defects and skin erosions characteristic of AEC syndrome. A chemical analog of Doxorubicin, diMe-Doxorubicin, which exhibits lower tissue and organ toxicity, was also found to be effective in promoting the disaggregation of mutant p63 and rescuing its transcriptional activity. Our findings identify compounds that can partially resolve mutant p63 aggregation, increase its monomeric isoform, and reactivate its transcriptional function. These results suggest potential therapeutic efficacy for treating skin erosions in AEC syndrome.

## Introduction

The p53 family member gene *TP63* is a key regulator of epidermal development and it is required for simple epithelial cells to commit to a stratified epithelial lineage during development [1–3] Due to the use of two independent transcriptional start sites (TA and ΔN) and differential splicing events at the 3′ UTR of its mRNA, at least six different transcripts are produced by *TP63* gene [1]. Among them, ΔNp63α protein is mostly found in the basal layers of stratified epithelia such as the epidermis, where it plays a crucial role in maintaining the self-renewing capacity of progenitor cells thus regulating keratinocyte proliferation and differentiation [4–6] The oligomerization domain (OD) allows p63 to work as a homo-tetramer, which is necessary to bind to the DNA with high affinity, or to form hetero-tetramer with p73 [7]. Furthermore, the C-terminus of the α isoforms includes a sterile-α-motif (SAM) domain, which is required for protein-protein interaction, and a trans-inhibitory domain (TID) which in turn carries a transcription inhibitory (TI) sequence.

A group of heterozygous mutations, that mostly clustered in the C-terminal domain, are frequently the cause of Ankyloblepharon-ectodermal dysplasia-cleft lip/palate (AEC, Hay-Wells syndrome, OMIM 106260) syndrome [8, 9] AEC syndrome is a rare ectodermal dysplasia, characterized by the abnormal development of ectodermal tissues including the skin, hair, nails, teeth, and sweat glands [10]. Among the most common features are potentially severe, long-lasting skin erosions, leading to chronic infection and scarring within the scalp, neck, hands, and feet. AEC inheritance follows a dominant autosomal pattern, with either missense mutations in the SAM domain (e.g., L514F), and less frequently in the TI domain (e.g., R598L, D601V), or as single-base frameshift mutations that cause the C-terminal domain to elongate (e.g., 3′ss intron 10, 1456InsA,1709DelA, 1859DelA) [11]. AEC mutations result in abnormal aggregation and abrogate p63 activation/repression of specific p63 target genes, including FGFR2 (fibroblast growth factor receptor 2) and KRT14 (keratin 14), resulting in impairment of expansion of epidermal progenitors and leading to the clinical manifestations of AEC [11]. The loss of function is an indirect result of aggregation rather than an intrinsic characteristic of the AEC mutants. This was demonstrated by the insertion of one or more mutations that decreased the aggregation tendency of AEC mutants, thereby restoring their transcriptional activity [11]. Therefore, a therapeutic approach could be aimed at a functional rescue through the treatment with small drugs that can prevent or restore the aggregation of AEC mutants. Here we performed a high-throughput screen using a luciferase reporter construct responsive to p63 in a search for small compounds that may recover mutant protein function. The screening showed the effectiveness of the widely used anthracyclines doxorubicin (Doxo) and epirubicin (Epi). While the anthracycline antibiotic family comprises hundreds of analogs, only a few are in clinical use, with Doxo and Epi being used against several forms of lymphoma, leukemia, sarcoma, and solid organ cancers [12, 13] The cytotoxicity of anthracyclines is mainly attributed to the interference with topoisomerase activity, induction of double-stranded DNA breaks (DSBs) and histone removal from transcriptionally active chromatin [14–16] Here we report that the aggregation of mutant p63 was alleviated in the presence of both Doxo and Epi in a heterologous system as well as in keratinocytes derived from the conditional knock-in mouse model for AEC syndrome. The ability of Epi and Doxo to disaggregate p63 may explain their ability to induce p63 activation. In addition, our finding showed that N,N-dimethyldoxorubicin (diMe-Doxo), an anthracycline analog which prevents cardiotoxicity [17], retains its ability to induce p63 mutant disaggregation. Our findings pave the way for the development of a novel anthracycline-based treatment for AEC syndrome.

## Results

### The anthracyclines Doxo and Epi identified by HTS are able to rescue mutant p63 transactivation function

**Fig. 1:**
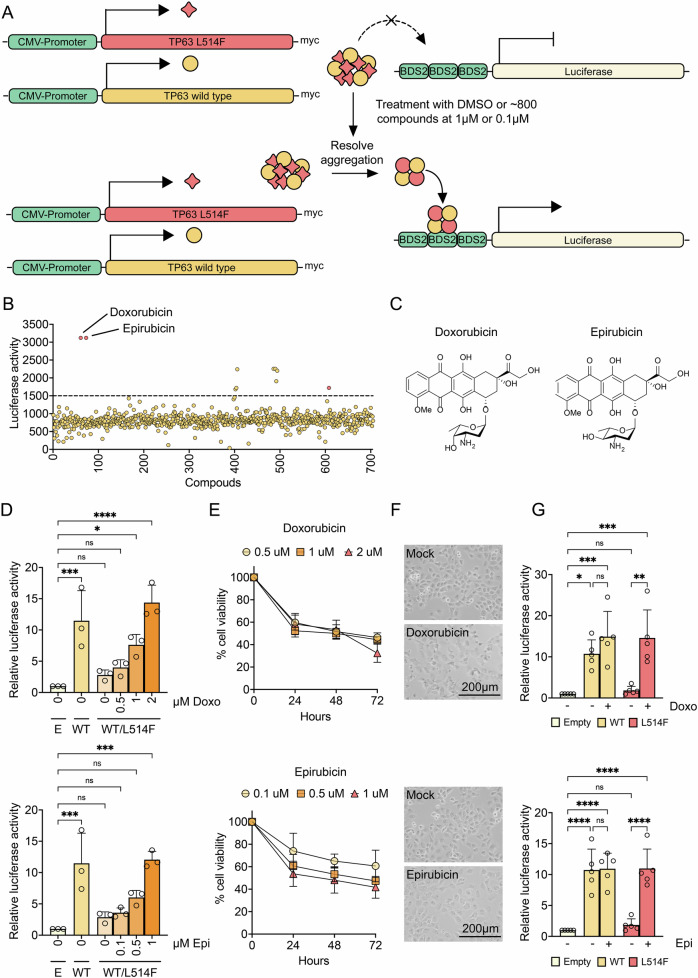
High-throughput screening (HTS) to identify small molecules that rescue mutant p63 transactivation function. **A** Schematic of the HTS based on luciferase reporter assay. H1299 cells were co-transfected with wild-type p63 and mutant L514F. Upon treatment with the FDA-approved drugs at 1 μM or with epigenetics compounds at 0.1 μM for 24 h, luciferase assay was performed. BDS2 3X promoter-luc was used as a reporter of p63 transcriptional activity. **B** Scatter plot of HTS results. A total of ~700 FDA-approved drugs were screened. Arbitrary cut-off threshold was set at a level 1500 of luciferase activity. Data points represent the average value of luciferase activity for each compound. The plate was run in triplicate. Statistically significant compounds are indicated in red. **C** Molecular structures of doxorubicin (Doxo) and epirubicin (Epi). **D** Luciferase reporter assay in H1299 cells co-transfected with a combination of wild-type p63 and mutant L514F, wild-type p63 alone or empty vector. 24 h after transfection, cells were incubated at the indicated concentrations of Doxo (upper) or Epi (lower) for 24 h and then analyzed for luciferase activity. Relative luciferase activity was normalized to transfection with empty vector. Data are represented as mean ± SD values (*n* = 3), *p < 0.05, **p < 0.01, ***p < 0.001, ****p < 0.0001, one way ANOVA test. **E** MTT assay was performed after treatment with the indicated concentration of Dox (upper) or Epi (lower) for 24, 48 and 72 h. Data are represented as mean ± SD values (*n* = 3). **F** Representative bright field images of H1299 cells after the treatment with 2 μM Doxo (upper) or 1 μM Epi (lower) for 24 h. Scale bar 200 *μ*m. **G** Luciferase reporter assay in H1299 cells transfected with wild-type p63, mutant L514F, or empty vector. 24 h after transfection, cells were incubated with 2 μM Doxo (upper) or 1 μM Epi (lower) for 24 h and analyzed for luciferase activity. Relative luciferase activity was normalized to transfection with empty vector. Data are represented as mean ± SD values (*n* = 5), *p < 0.05, **p < 0.01, one way ANOVA test.

Our recent findings indicate that both missense and frameshift mutations underlying AEC syndrome elicit p63 protein aggregation, thereby acting in a dominant-negative fashion to disrupt p63 tetramer function [11] (Fig. S1A). In order to develop a high-throughput screening (HTS) strategy in a search for small compounds that may disentangle mutant p63, we tested the ability of different reporter constructs to drive the expression of the luciferase gene in transiently transfected H1299 cells, that are devoid of p53 and its family members. The three different reporter constructs, respectively, carry the firefly luciferase gene under the control of Keratin 14 (KRT14) promoter, fibroblast growth factor receptor 2 (FGFR2) enhancer, and BDS2 3X, with the latter being a p53-responsive element that is likewise highly responsive to p63 (Fig. S1B). The same amount of wild-type p63, as shown by western blot, efficiently activated the BDS2 3X and KRT14 promoters leading to a nine-fold and five-fold increase in luciferase activity respectively, whereas the use of the FGFR2 enhancer was not associated with statistically significant induction (Fig. S1B). We next examined the responsiveness of the BDS2 3X reporter construct in cells overexpressing AEC-associated p63 mutants, with or without the V603D mutation. The V603D mutation restores transcriptional activity in the p63 mutants and eliminates protein aggregation of p63 with AEC mutations that occur in the TI domain, such as the R598L and D601V mutations, but not those that occur in the SAM domain, such as the L514F mutation [11]. As previously demonstrated in HEK293 cells [11], all AEC mutants exhibited decreased luciferase activity, whereas the V603D variant completely restored the transactivation function of the mutants R598L and D601V, and to a lesser extent, as expected, of the mutant L514F (Fig. S1C). Furthermore, AEC mutants have been demonstrated to inactivate wild-type p63 through coaggregation [11]. This was confirmed in our experiments in which the different mutant forms of p63 were overexpressed in heterozygous or homozygous states in H1299 cells. Luciferase activity was strongly reduced in homozygous conditions and to a lesser extent in heterozygous conditions compared with wild-type p63 control (Fig. S1D–F). Thus, after confirming the feasibility of the HTS using the BDS2 3X-Luc reporter construct, two chemical libraries, including ~700 FDA-approved drugs and 88 epigenetic compounds, were screened in H1299 cells co-transfected with wild-type p63 and the L514F mutant to best mimic the heterozygous nature of the disorder. We hypothesize that compounds able to recover the aggregation of mutant p63 would result in the measurable activity of the luciferase reporter (Fig. 1A). Ten FDA-approved drugs and six epigenetics compounds were defined as positive hits based on the arbitrary cut-off threshold set at a level 1500 and 3000 of luciferase expression induction, respectively (Fig. 1B and Fig. S2A). Afterward, the 6 compounds which showed a statistically significant induction were further examined in their ability to induce in a dose-dependent manner the luciferase activity in H1299 cells. Among these, only two compounds belonging to the anthracycline family, doxorubicin (Doxo) and its epimer epirubicin (Epi), were able to rescue p63 transactivation function at comparable level to wild-type p63 and showing a dose-dependent induction of luciferase activity in H1299 cells (Fig. 1C, D and Fig. S2B–E). The most prominent induction was found using 2 μM Doxo and 1 μM Epi.

Anthracyclines are topoisomerase II poisons used as chemotherapeutic treatments for various types of cancer which exert their action through histone eviction and causing double-stranded breaks (DSBs) [12, 13]. Therefore, Doxo and Epi were examined for their toxicity by using an MTT cell viability assay. Both compounds decreased the viability of H1299 cells in a concentration-dependent manner (Fig. 1E), although 1 μM Epi appeared to affect less the cell morphology compared to 2 μM Doxo (Fig. 1F). We next evaluated whether the most effective concentration of the compounds had the ability to recover p63 transcription activity also in the presence of only the mutant form. Likewise, the treatment with 2 μM Doxo and 1 μM Epi promoted the rescue of luciferase activity induction to a similar level to wild-type p63 (Fig. 1G). Although these compounds have some toxic effects on H1299 cells, both Doxo and Epi were able to reactivate mutant p63 independently of the presence of p63 wild-type.

### Doxo and Epi induce disaggregation of mutant p63 protein

**Fig. 2:**
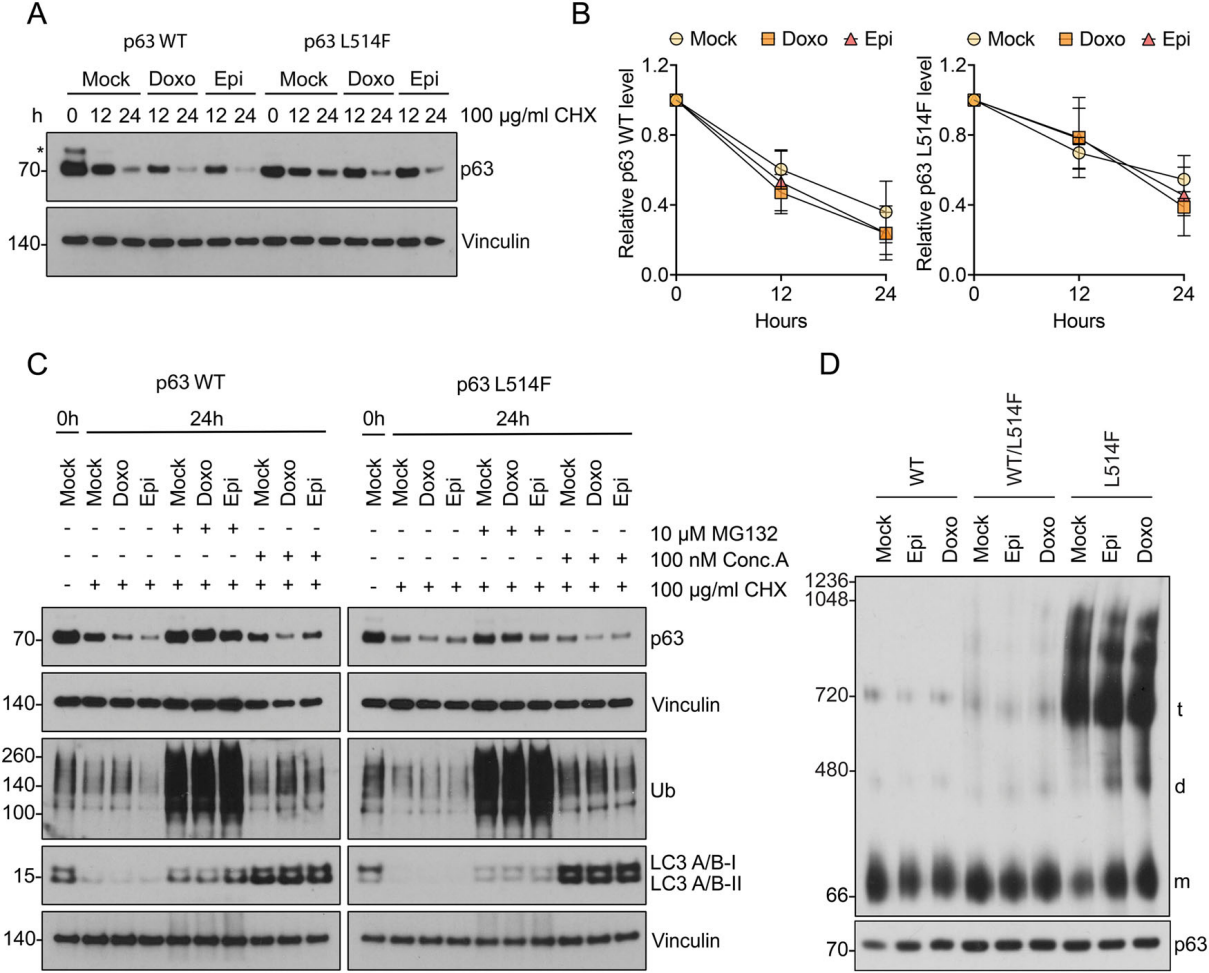
Doxo and Epi induce disaggregation of p63 L514F protein in H1299 cells. **A** SDS-PAGE followed by Western blot for p63 in H1299 cells transfected with wild-type p63 and mutant L514F. 24 h after transfection, cells were treated with 100 μg/mL CHX and incubated with 2 μM Doxo or 1 μM Epi. At the indicated time points, cells were harvested, lysed, and analyzed for p63 levels. The asterisk indicates a non-specific band. Vinculin levels were measured as a loading control. **B** Quantitative analysis of the p63 protein levels in cells after CHX treatment. Data are represented as mean ± SD (*n* = 3). **C** SDS-PAGE followed by Western blot for p63 in H1299 cells transfected with wild-type p63 or mutant L514F. 24 h after transfection, cells were treated with 100 μg/mL CHX, a combination of 100 μg/mL CHX and 10 μM MG132 to inhibit the proteasome, or a combination of 100 μg/mL CHX and 100 nM Concanamycin A to inhibit autophagy. Cells were incubated with 2 μM Doxo or 1 μM Epi for 24 h. Cells were harvested and analyzed for p63 levels. As controls of treatment efficiency, LC3 A/B-I and II protein level increased when autophagy was inhibited, and antibodies anti-Ubiquitin revealed an accumulation of poly-ubiquitinated proteins when the proteasome was blocked. Vinculin levels were measured as a loading control. **D** Blue-native (BN) PAGE followed by Western blot for p63 in H1299 cells transfected with mutant L514F alone or together with wild-type p63 and treated with 2 μM Doxo or 1 μM Epi for 48 hr. Samples were normalized for p63 amount by Western blot analysis. m monomer, d dimer, t tetramer.

To investigate the mechanism by which Doxo and Epi restore p63 transactivation function, we evaluated whether these compounds promote degradation or disaggregation of AEC-associated p63 mutants. To test the hypothesis that Doxo and Epi influence the degradation of mutant p63 protein, H1299 cells were transfected with wild-type p63 or the L514F mutant, followed by treatment with cycloheximide (CHX) to inhibit protein synthesis. SDS-PAGE followed by Western blot for p63 revealed that these compounds promote the degradation not only of the mutant form of the protein but also of wild-type p63 (Fig. 2A, B). We questioned whether the degradation of the wild-type and mutant proteins induced by anthracyclines occurs via distinct degradation pathways. Autophagy and the ubiquitin-proteasome system (UPS) are the two major pathways for protein degradation [18]. It has been reported that anthracyclines can act on the ubiquitin-proteasome system inducing the degradation of specific targets [19, 20]. To investigate whether p63 degradation occurs by autophagy or by the proteasome, we treated H1299 cells with CHX alone, a combination of CHX and MG132 to also inhibit the proteasome, or a combination of CHX and Concanamycin A to inhibit autophagy. SDS-PAGE followed by Western blot analysis for p63 revealed that the levels of both wild-type and mutant p63 protein were restored by proteasome inhibition (Fig. 2C) while no significant effect was observed when autophagy was inhibited. However, when cells were treated with Dox and Epi in the presence of MG132, the downregulation of p63 protein was not significantly blocked. These results suggest that while the proteasome system primarily mediates p63 degradation, Doxo and Epi do not significantly affect this event for mutant p63. Therefore, we considered the possibility that these compounds may have a higher impact on inducing mutant p63 disaggregation. To examine the capacity of Doxo and Epi to induce mutant p63 disaggregation, BN-PAGE followed by Western blotting for p63 was performed in H1299 cells lysates transfected with wild-type p63, mutant L514F, or a combination of wild-type and mutant p63 to simulate the heterozygous state. As expected, wild-type p63 ran primarily as a monomer, while p63L514F overexpression resulted in the formation of large multimeric assemblies. Doxo and Epi were able to reduce aggregation and increase the formation of monomeric form in H1299 expressing p63L514F in homozygous states (Fig. 2D). To summarize, we found that Doxo and Epi do not preferentially promote the degradation of mutant p63 protein in H1299 cells but interestingly they seem to specifically affect its disaggregation.

### Epi induces disaggregation of p63 L514F protein and rescues mutant p63 transcriptional activity in primary keratinocytes derived from the skin of a AEC mouse model

**Fig. 3:**
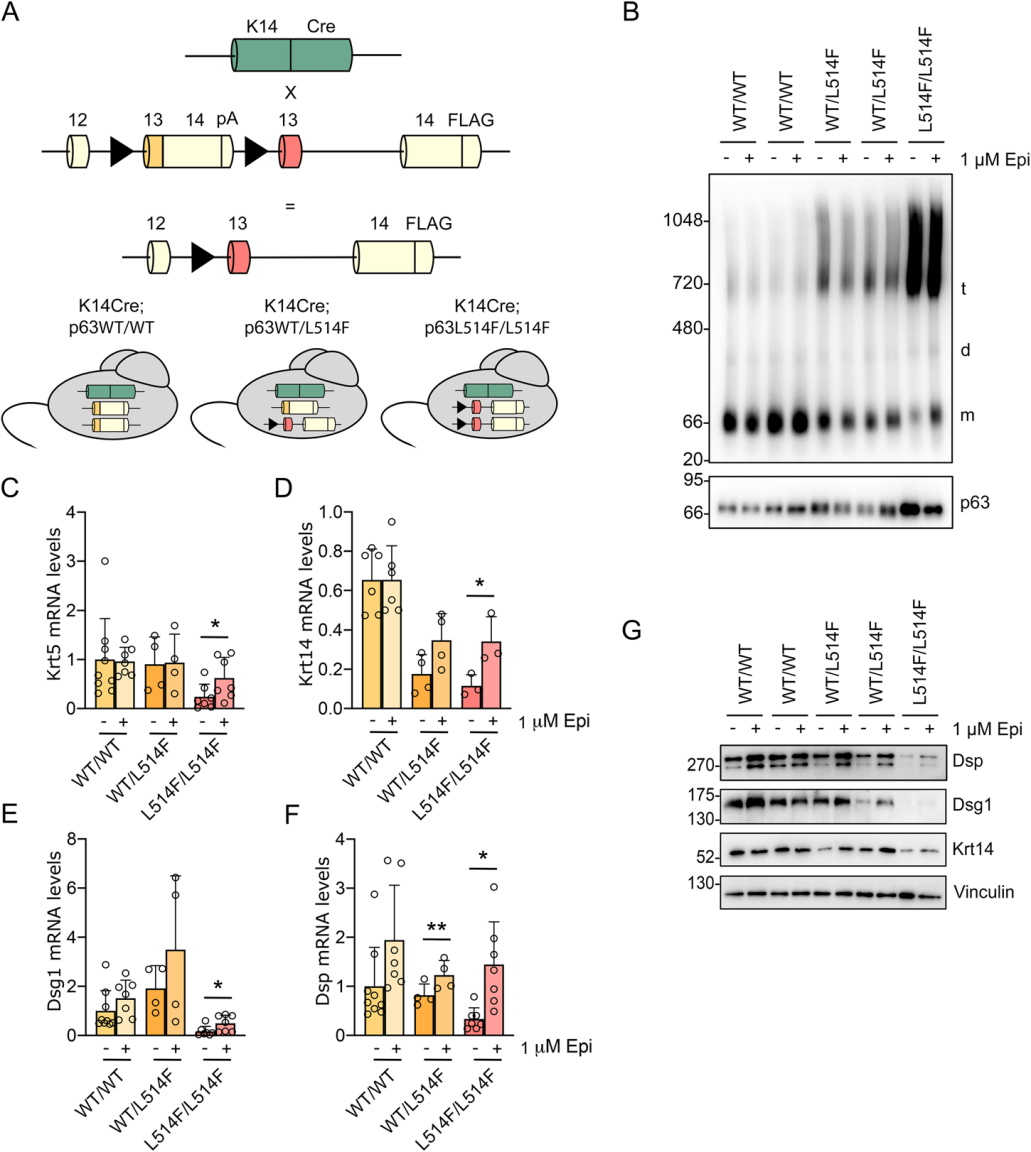
Epi restores mutant p63 transcriptional activity in mouse primary keratinocytes. **A** Schematic strategy for the generation of p63^+/L514Fflox^ knock-in mice [11]. Mutant exon 13 carrying the L514F mutation is indicated in red. LoxP sites (black triangles) flank wild-type exon 13 (yellow) fused with the coding portion of exon 14 and the SV40 polyA (pA). 3xFLAG was placed at the end of the coding sequence in exon 14. FLAG-tagged p63 mutant protein is expressed upon Cre mediated deletion. **B** BN-PAGE followed by p63 Western blot of primary keratinocyte lysates isolated from mice with the indicated genotypes. Cells were treated with 1 μM Epi for 36 h. After treatment, cells were harvested, lysed and analyzed for p63 levels. Samples were normalized for p63 amount by Western blot analysis. m monomer, d dimer, t tetramer. **C**–**F** Real-time RT-PCR analysis of the keratinocyte-specific p63 target genes Krt5, Krt14, Dsg1, and Dsp in primary keratinocytes following the treatment with 1 μM Epi for 36 h. Data are represented as mean ± SD (WT *n* > 6; WT/L514F *n* = 4; L514F/L514F *n* > 3). *p < 0.05, **p < 0.01, paired two-tailed *t*-test. **G** SDS-PAGE followed by Western blot for the protein products of p63 target genes in primary keratinocytes isolated from mice with the indicated genotypes following the treatment with 1 μM Epi for 36 h. Vinculin was used as a loading control.

To test these compounds in a more patho-physiological setting, we took advantage of the conditional knock-in mouse model previously generated in our laboratory in which the AEC mutation L514F in exon 13 is expressed only in the presence of the Cre recombinase under the control of the endogenous Krt14 promoter (Fig. 3A) [11]. K14-Cre; p63^+/L514Fflox^ newborn mice were indistinguishable from wild-type mice and they did not reflect the severity of the human disorder, while Krt14-Cre; p63^L514Fflox/L514Fflox^ mice fully recapitulate the skin defects and erosions found in AEC patients. Since in mouse primary keratinocytes Epi had a reduced toxic effect as compared to Doxo (Fig. S3A, B), keratinocytes isolated from the conditional knock-in model were treated with Epi only and then protein extracts were subjected to BN-PAGE followed by Western blotting for p63. As expected, protein aggregation was detected in primary keratinocytes derived from Krt14-Cre;p63^+/L514Fflox^ and Krt14-Cre;p63^L514Fflox/L514Fflox^ mice, but not in wild-type cells (Fig. 3B). While p63 from wild-type keratinocytes mainly runs as a monomer, mutant keratinocytes exhibit severe aggregation and reduced monomeric p63. Consistently with the results observed in H1299 cells, aggregation was progressively alleviated in the presence of Epi in mutant keratinocytes, accompanied by the increase of the monomeric form of p63 (Fig. 3B).

Since it was previously demonstrated that critical p63 target genes were significantly downregulated in keratinocytes derived from K14-Cre; p63^L514Fflox/L514Fflox^ mice [11], we tested the hypothesis that by reducing protein aggregation via Epi treatment, mutant p63 transcriptional function may be at least partially restored. Thus, the ability of p63wt and p63L514F to upregulate keratinocyte-specific p63 target genes, including keratin 5 (Krt5), keratin 14 (Krt14), and desmosomal genes such as desmoglein 1 (Dsg1) and desmoplakin (Dsp), was tested after treatment with Epi. Real-time RT-PCR analysis confirmed transcriptional rescue of keratinocyte-specific p63 target genes after Epi treatment (Fig. 3C–F). p63 target genes affected in AEC syndrome were induced in Epi-treated mutant and heterozygous keratinocytes compared to untreated controls, confirming that this compound can rescue mutant p63 transcriptional activity. Since Epi partially rescued the transcriptional activity of mutant p63, we tested if it could also recover the protein expression of p63 targets. Protein extracts were analyzed by SDS-PAGE followed by immunoblotting with antibodies against Krt14, Dsp, and Dsg1, showing expression of p63 targets to be severely affected in mutant keratinocytes, compared to wild-type cells (Fig. 3G). Interestingly a partial rescue was observed for the desmosome proteins and Krt14 after the treatment with Epi. Together these findings demonstrated that, since Epi alleviate aggregation, it is able to induce a transcriptional rescue of mutant p63.

### The less cardiotoxic anthracycline analog N,N-dimethyldoxorubicin is still effective in recovering mutant p63 transcriptional activity

**Fig. 4:**
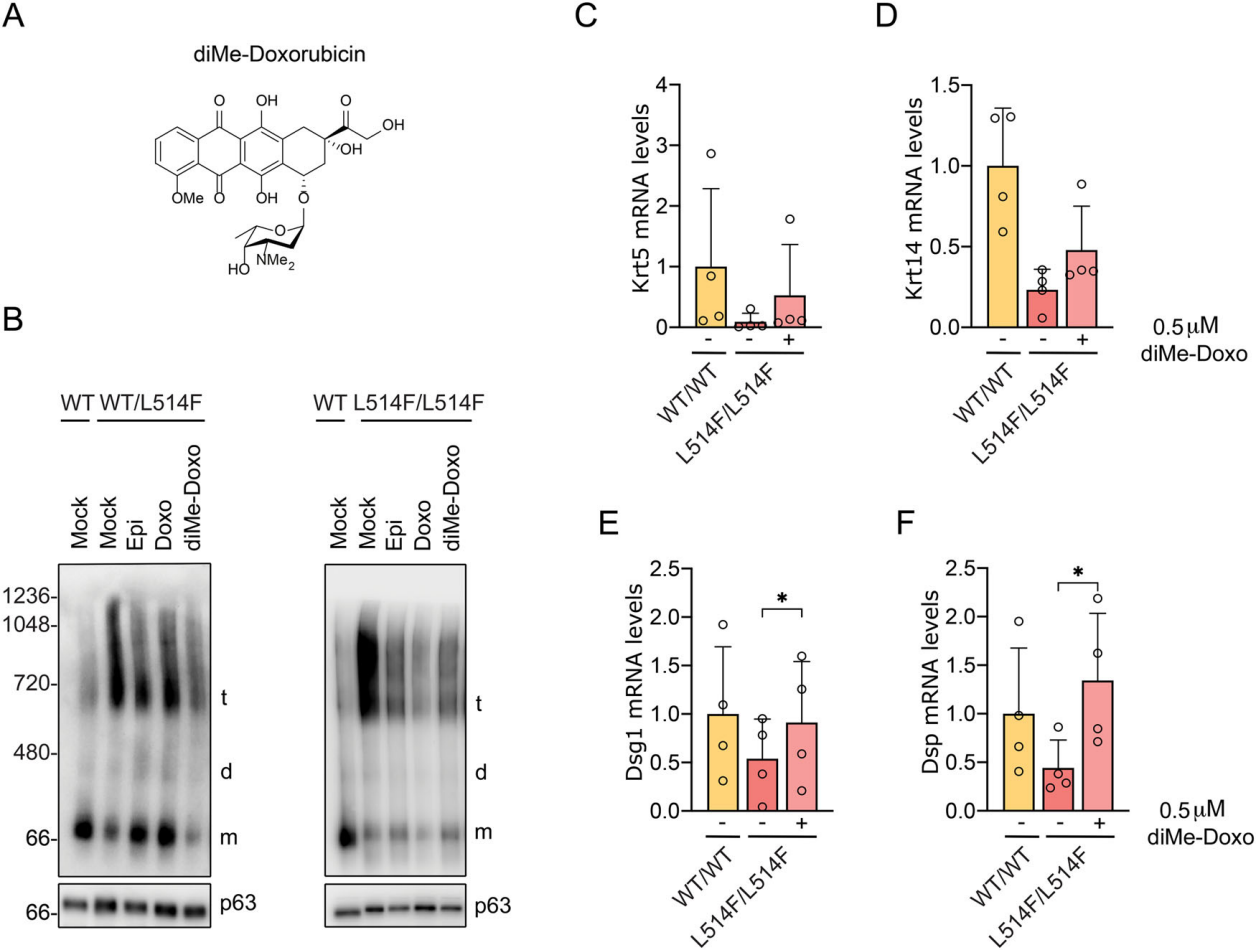
diMe-Doxo is able to rescue mutant p63 transcriptional activity. **A** Molecular structures of diMe-Doxo. **B** BN-PAGE followed by p63 Western blot of primary keratinocyte lysates isolated from mice with the indicated genotypes. Cells were treated with 1 μM Epi, 1 μM Doxo or 0.5 μM diMe-Doxo for 36 h. After treatment, cells were harvested, lysed and analyzed for p63 levels. Samples were normalized for p63 amount by Western blot analysis. m monomer, d dimer, t tetramer. **C**–**F** Real-time RT-PCR analysis of the keratinocyte-specific p63 target genes Krt5, Krt14, Dsg1, and Dsp in primary keratinocytes following the treatment with the indicated compounds for 36 h. Data are represented as mean ± SD (WT *n* = 4; L514F/L514F *n* = 4). *p < 0.05, paired two-tailed *t*-test.

Topoisomerase II inhibitors such as Doxo and Epi are associated with enduring cardiotoxic effects, which limit their clinical usage in cancer treatment [21, 22]. In a recent study, the anthracycline analog N,N-dimethyldoxorubicin (diMe-Doxo) was synthesized to reduce the toxicity of Doxo by chemically removing the DNA-damaging effect (Fig. 4A and Fig. S4A) [17]. Indeed, diMe-Doxo demonstrated lower toxicity and reduced cardiotoxicity in mice [17]. Taking into account the benefits of this chemical variant, we sought to determine whether diMe-Doxo may be used as a compound to promote p63 mutant protein disaggregation. In line with previous findings [17], the impact of diMe-Doxo on cell viability is comparable to the other anthracyclines already evaluated (Fig. S4B, C). To address whether diMe-Doxo retains the ability of the previously tested anthracyclines to disaggregate mutant p63 protein, we performed BN-PAGE followed by Western blotting for p63 in mouse primary keratinocyte lysates from the mouse conditional knock-in model for AEC syndrome treated with Epi, Doxo and diMe-Doxo. The p63 protein aggregate detected in primary keratinocytes derived from Krt14-Cre;p63^+/L514Fflox^ and Krt14-Cre;p63^L514Fflox/L514Fflox^ mice was progressively reduced in the presence of diMe-Doxo (Fig. 4B). We then evaluated the recovery of the transcriptional activity of p63 L514F of different target genes after the treatment with diMe-Doxo. Consistent with its disaggregation ability, diMe-Doxo was able to restore transcriptional activity of mutant p63 promoting the upregulation of p63 target genes (Fig. 4C–F). These results indicate that diMe-Doxo, which presents less cardiotoxic effects [17], still efficiently induces mutant p63 disaggregation, thereby promoting the rescue of its transcriptional function.

## Discussion

Heterozygous mutations in p63 gene can cause different rare genetic disease including AEC syndrome, characterized by skin fragility, severe long-lasting skin erosions and orofacial defect [23, 24]. A combination of surgical repair for oral clefting abnormalities and other dental procedures can correct a variety of defects. However, skin erosions remain a significant unmet medical need, because they are often difficult to treat. Delayed and inefficient wound healing puts patients at risk for secondary infections. Therefore, there is an urgent need to develop targeted therapeutic approaches to improve skin integrity in these patients. AEC-associated p63 mutants display an impaired transactivation activity as a consequence of protein structural abnormalities which cause a decrease in p63 binding ability for DNA. Reducing the aggregation tendency of AEC mutants through deletion of aggregating peptides or introduction of specific amino acid substitutions lead to the rescue of p63 transcriptional function [11]. Thus, in this study we designed a functional high-throughput assay to screen small compounds that could counteract aggregation of mutant p63 recovering its transactivation activity in cells co-expressing wild-type p63 and its mutant form p63L514F. HTS approaches that utilize small molecule libraries composed of known compounds offer a valuable opportunity to identify candidates with well-characterized on- and off-target effects, established toxicology profiles, and, in many cases, existing data on human use. This approach enables a more streamlined and efficient pathway for drug repurposing, which holds significant potential for addressing unmet medical needs. By leveraging known compounds, researchers can bypass many of the lengthy and costly steps associated with de novo drug development, such as early-stage safety assessments and dosage optimization. Consequently, this strategy can substantially shorten the time and reduce the expenses required to bring effective therapies to patients, accelerating the overall drug discovery and approval process. From the results of the HTS, two compounds were isolated that were able to rescue mutant p63 transactivation ability, Doxo and Epi, that are antineoplastic antibiotic from the anthracycline family. In a quest to determine how anthracyclines resolve the aggregation of mutant p63, we observed that anthracyclines did not have a specific effect on its degradation. Indeed, Doxo and Epi accelerated the degradation of both wild type and mutant proteins. Our data suggested that the main pathway responsible of p63 degradation is the ubiquitin-proteasome system. However, when cells were co-treated with Doxo and Epi and the proteasome inhibitor MG132, the degradation of p63 protein was not substantially prevented. Recent findings have revealed that DNA-damaging agents, such as Doxo, promote the ISG15 conjugation (ISGylation) of the p63 protein [25, 26]. ISG15 is a ubiquitin-like protein (UBL) which can regulate protein stability by either enhancing or suppressing the degradation of ISGylated proteins through the UPS or lysosomes [27–29]. Unlike other UBLs that are constitutively expressed, ISGylation is mainly induced by the presence of interferon IFNs but also by various cell perturbations such as DNA damage [30, 31]. It is intriguing to speculate that both Doxo and Epi may enhance p63 ISGylation, which in turn could promote the degradation of p63. However, rather than promoting degradation, anthracyclines specifically influenced the disaggregation of mutant p63 in H1299 cells. We found that in H1299 extracts expressing p63 L514F or a combination of p63wt and p63L514F the aggregation of mutant protein was alleviated in the presence of the compounds and the same effect was observed also in murine keratinocytes where p63 is endogenously expressed. Consistently, target genes positively regulated by p63 and affected in AEC syndrome, including Krt14, Krt5, Dsp and Dsg1, were induced in mutant keratinocytes treated with Epi confirming that transcriptional function of p63 may be at least partially recovered by decreasing protein aggregation by treatment with the compound.

The use of anthracyclines is limited because of the well-established adverse effects, among which cardiotoxicity is the primary one that limits treatment [21, 22]. In the past several years, several anthracycline analogs have been examined in an effort to find anthracyclines with fewer side effects [32, 33]. Recently, the anthracycline analog diMe-Doxo was synthesized; this chemical variant maintains chromatin remodeling activity but does not generate DNA damage, typical characteristic of anthracycline family. diMe-Doxo has been demonstrated to be less toxic and to mitigate cardiotoxicity in mice as opposed to Doxo [17]. Here we report that diMe-Doxo is still effective in causing mutant p63 disaggregation, which aids in the restoration of its transcriptional function. The ability of diMe-Doxo to induce p63 disaggregation, despite lacking DNA-damaging activity, indicates that p63 disaggregation may be driven by an independent mechanism, not necessarily linked to the DNA damage response. In conclusion, our study suggests that anthracyclines may serve as a promising starting point for developing a therapy aimed at restoring p63 activity by targeting the aggregation propensity of AEC-associated p63 mutant proteins.

Although the anthracyclines examined in this study are known to have adverse side effects, future research should focus on secondary screening to identify chemical variants that retain therapeutic activity but exhibit reduced toxicity. This approach could lead to the development of a therapy that significantly improves the health outcomes of patients with AEC syndrome.

## Materials and methods

### Mice

K14-Cre (Krt14-CreΔneo) knock-in mice were obtained from J. Huelsken,Swiss Institute for Experimental Cancer Research, Lausanne, Switzerland, and were used to induce expression of the p63 mutant protein. All mouse work was conducted at CEINGE according to the Italian ethical regulations under the animal license 928/2021-PR.

### Mouse genotyping

Mouse genotyping was performed by PCR starting from mouse tail DNA. The oligonucleotides primers used for genotyping were:

FloxL514F Fw (5’-CAGCGTATCAAAGAGGAAGGAGA)

FloxL514F Rv (5’-AGCCAGAATCAGAATCAGGTGAC)

Cre-recombinase Fw (5’-GGCAGTAAAAACTATCCAGCAACA)

Cre-recombinase Rv (5’-TAACATTCTCCCACCGTCAGTA)

### Cell culture

Primary mouse keratinocytes were isolated from newborn skin as previously described [34] and grown at 34 °C and 8% CO2 in low calcium medium (0.05 mM CaCl2) supplemented with 4% of calcium-chelated fetal bovine serum (FBS) and epidermal growth factor (EGF).

H1299 cells were maintained in Dulbecco’s Modified Eagle Medium (DMEM) supplemented with 10% FBS. Cells were grown at 37°C in a humidified atmosphere of 5% (vol/vol) CO2.

### Plasmid DNA and transfection methods

PCR-generated inserts were subcloned into pcDNA3.Myc to produce pcDNA3.Myc-ΔNp63α. Site-directed mutagenesis was then used to obtain all *p63* mutant constructs [11]. Cells were transiently transfected using Lipofectamine 2000 (Thermo Fisher Scientific).

### Reagents

Doxo (D1515) and Epi (E9406) were purchased from Sigma-Aldrich. diMe-Doxo was provided by Dr. Jacques Neefjes. Cycloheximide (C7698), MG-132 (M7449) and Concanamycin A (C9705) were purchased from Sigma-Aldrich. All reagents were dissolved according to the manufacturer’s formulation. The FDA-approved drugs library was purchased from Selleckem (Cat. No. L1300). The epigenetics screening library was purchased from Cayman Chemical Company (Cat. No. 11076).

### Luciferase assay

The luciferase reporter assay was performed using a Luciferase Reporter Assay Kit (Promega) according to the manufacturer’s recommendations. H1299 cells were seeded in 24-well plates. pKRT14 promoter-luc [35], pFGFR2 enhancer-luc [36] and pBDS2 3X-luc [37] were used as reporters. Luminescence was determined 24 h or 48 h after transfection using EnVision plate reader.

### High-throughput screening

The screening of compounds was permorfed using a Luciferase Reporter Assay Kit (Promega). H1299 cells were seeded in 384-well plates and co-transfected with wild-type p63 and mutant L514F. pBDS2 3X-luc was used as reporter of p63 transcriptional activity. Upon 24 h treatment with the FDA-approved drugs at 1 μM (Selleckem, Cat. No. L1300) or with epigenetics compounds at 0.1 μM (Cayman Chemical Company, Cat. No. 11076), luciferase assay was performed and luminescence was determined using EnVision plate reader.

### MTT assay

H1299 or mouse primary keratinocytes cells were seeded into 96-well plates. 24 h after seeding, cells were treated with the indicated drugs for 24, 48, 72 h, at the indicated concentrations, followed by standard MTT assay (abcam). Cells were incubated for 3 h at 37 °C in the dark with media containing 0.5 mg/mL of MTT solution. Afterward, medium was replaced with isopropanol and incubated for another 30 min at 37 °C. Then absorbance at 570 and 620 nm was determined using EnVision plate reader.

### SDS-PAGE, blue native (BN)-PAGE, and western blot

For BN-PAGE, H1299 cells or mouse primary keratinocytes were scraped on ice in native lysis buffer (25 mM Tris (pH 7.5), 150 mM NaCl, 10 mM MgCl2, 10 mM EDTA (pH 8.0), 20 mM CHAPS, 2 mM DTT, protease and phosphatase inhibitors), then collected and incubated for 1 h in ice in the presence of benzonase (70746, Merck Millipore) for lysis and digestion of nucleic acids. Protein extracts were then mixed with 20% glycerol and 5 mM Coomassie G-250 (Thermo Fisher Scientific) and loaded on 3–12% Novex Bis-Tris gradient gels for BN-PAGE (Thermo Fisher Scientific), according to the product manuals, followed by western blotting. For SDS-PAGE, cells were lysed in LDS Sample Buffer 2X (Thermo Fisher Scientific), boiled and loaded on denaturing SDS-PAGE gels followed by western blotting. Membranes were blocked with PBS 0.2% Tween 20 in 5% nonfat-dry milk and incubated with primary antibodies for 2 h at room temperature (RT) or overnight at 4°C. The primary antibodies used for Western blot analysis were: anti-p63 EPR5701 (ab124762, Abcam), anti-vinculin (sc73614, Santa Cruz), anti-ubiquitin P4D1 (sc-8017, Santa Cruz), anti- LC3A/B D3U4C (12741, Cell Signaling), anti-Krt5 (905501, Biolegend), anti-Krt14 (905304, Biolegend), anti-Dsp 1–2 (61003, Progen Biotechnik GmbH), anti-Dsg1 B11 (sc-137164, Santa Cruz), and anti-γH2AX Ser139 (2577, Cell Signaling). Membranes were incubated for 1 h at RT with the appropriate horseradish peroxidase-conjugated secondary antibody and detected by chemiluminescence.

### RNA isolation and RT-qPCR

Total RNA was extracted from cells using TRIzol reagent (Thermo Fisher Scientific) and retro-transcribed to cDNA using High-Capacity cDNA Reverse Transcription Kit (Thermo Fisher Scientific). RT-qPCR was performed using Luna Universal qPCR Master Mix (New England Biolabs) in an ABI PRISM 7900 instrument (Thermo Fisher Scientific). Target genes were quantified using the following specific oligonucleotide primers and normalized to mouse β-actin expression:

ß-actin Fw (5’-CTAAGGCCAACCGTGAAAAGAT)

Rv (5’-GCCTGGATGGCTACGTACATG)

Krt5 Fw (5’-CAACGTCAAGAAGCAGTGTGC)

Rv (5’-TTGCTCAGCTTCAGCAATGG)

Krt14 Fw (5’-ACCACGAGGAGGAAATGGC)

Rv (5’-TGACGTCTCCACCCACCTG)

Dsp Fw (5’-CACCGTCAACGACCAGAACTC)

Rv (5’-GATGGTGTTCTGATTCTGATGTCTAGA)

Dsg1 Fw (5’-TCACCCCCTTTTTCATTATCTACTG)

Rv (5’-GTGGATTCTCCAAGTCTTGACCTT)

### ROS assay

Mouse primary keratinocytes cells were seeded into 96-well plates. 24 h after seeding, cells were treated with the indicated drugs for 24 h at the indicated concentrations followed by ROS Detection with ROS Detection Cell-Based Assay Kit (Cayman Chemical Company) according to manufacturer’s instructions. Cells were incubated with 10 μM of 2,7-Dichlorofluoroscin Diacetate (DCFDA) for 1 h at 37 °C in the dark. Fluorescence was measured using EnVision plate reader.

### Quantification and statistical analysis

All datasets derive from at least three independent experiments unless otherwise indicated. Data are presented as the mean of independent experiments ± SD as indicated. The number of independent experiments or the number of analyzed animals are indicated (n). All statistical analyses were performed using GraphPad Prism software (version 10.0). In experiments comparing two samples paired two-tailed *t*-testing was performed, whereas when comparing multiple independent samples, one-way analysis of variance (ANOVA) were performed as described in Figure legends. Western blot were quantified using ImageJ software. *P*-values of statistical significance are represented as *p < 0.05, **p < 0.01, ***p < 0.001, ****p < 0.0001.

## Supplementary information

Original DATA Western Blots

Supplementary Information

## Data Availability

All data support the findings of this study are included in the main text and supplementary information (SI). All procedures of experiments are described in detail in Materials and Methods.
