# Supplementary figures and images for: Anthracyclines disaggregate and restore mutant p63 function: a potential therapeutic approach for AEC syndrome
Source: Cell Death Discov. 2025 Jan 25;11:24. doi: 10.1038/s41420-025-02307-0 (PMC11762975; doi:10.1038/s41420-025-02307-0)

Figure 2

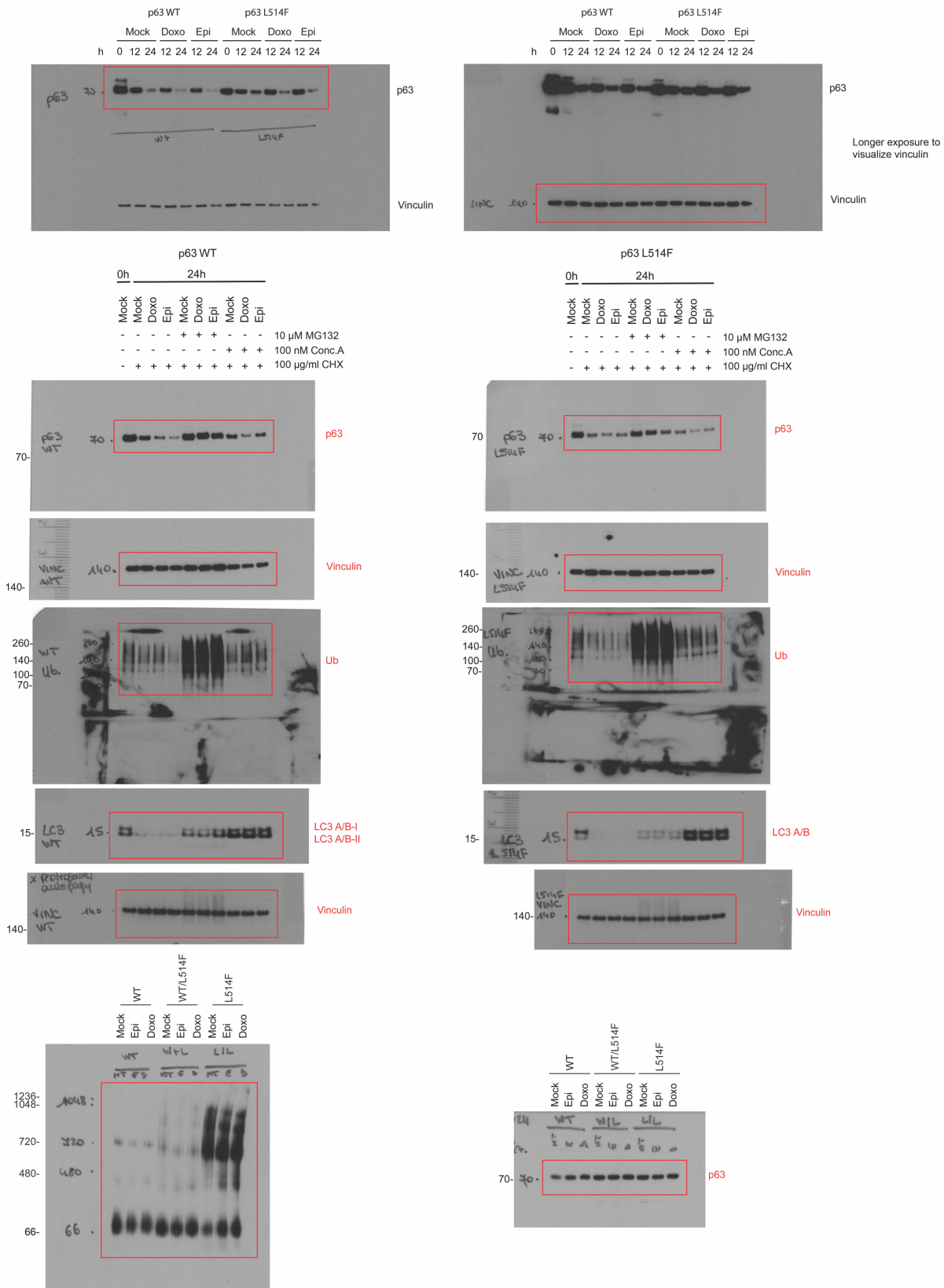

Figure 3

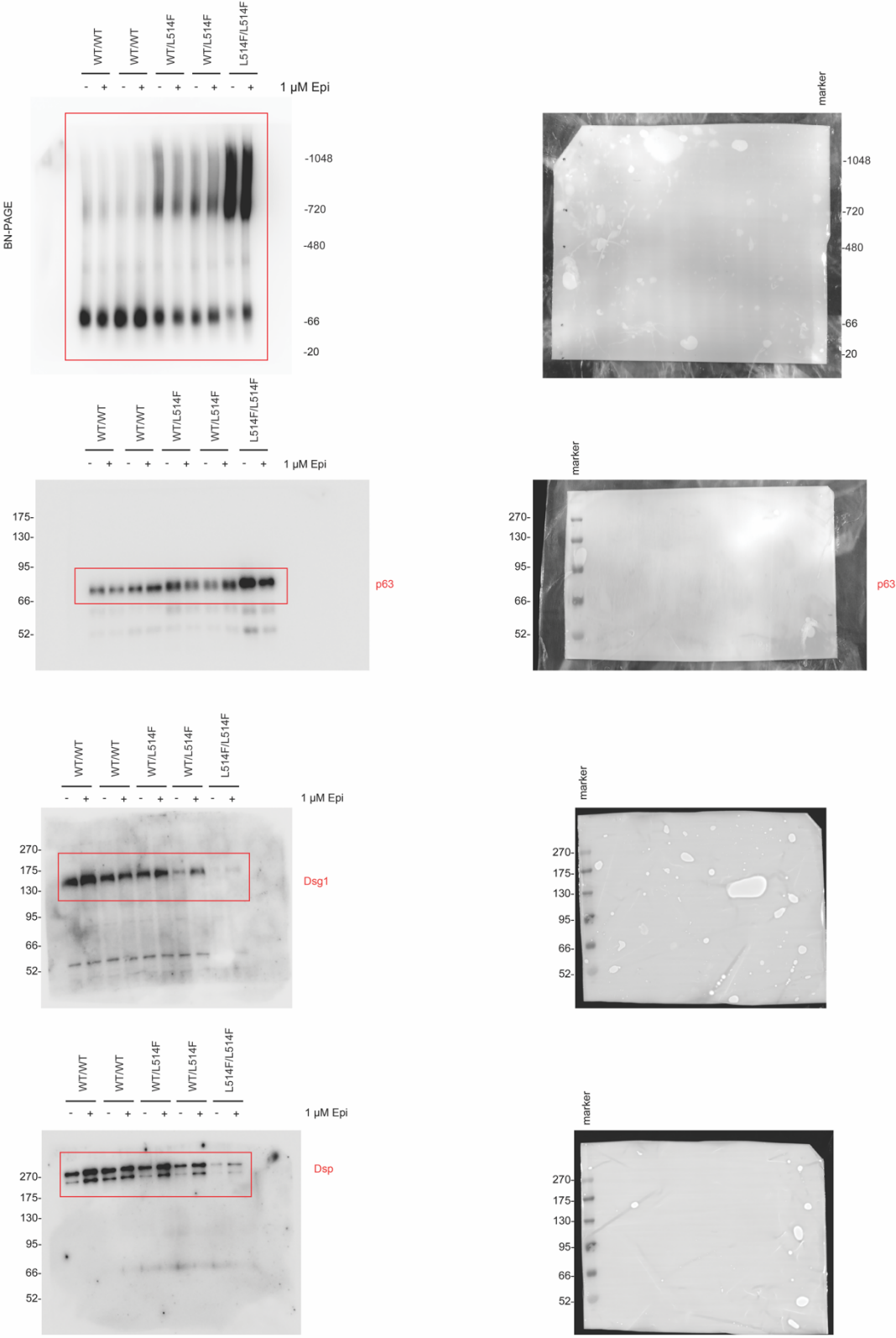

Figure 3 - Continued

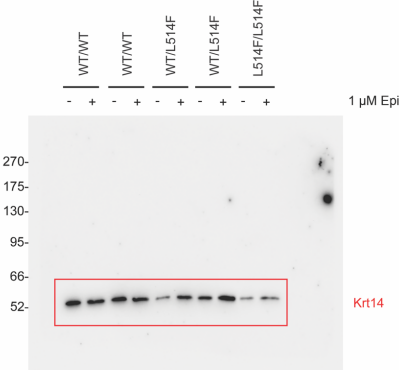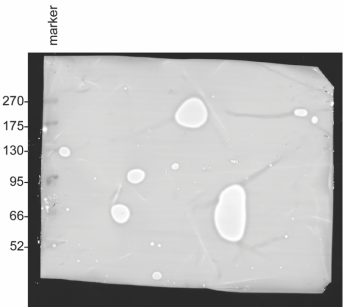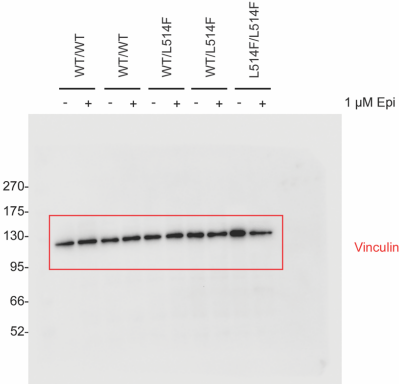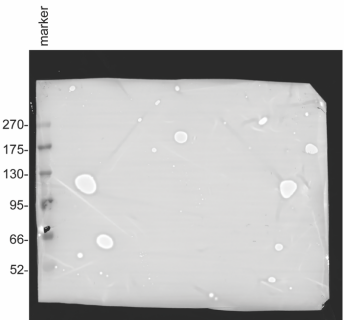

Figure 4

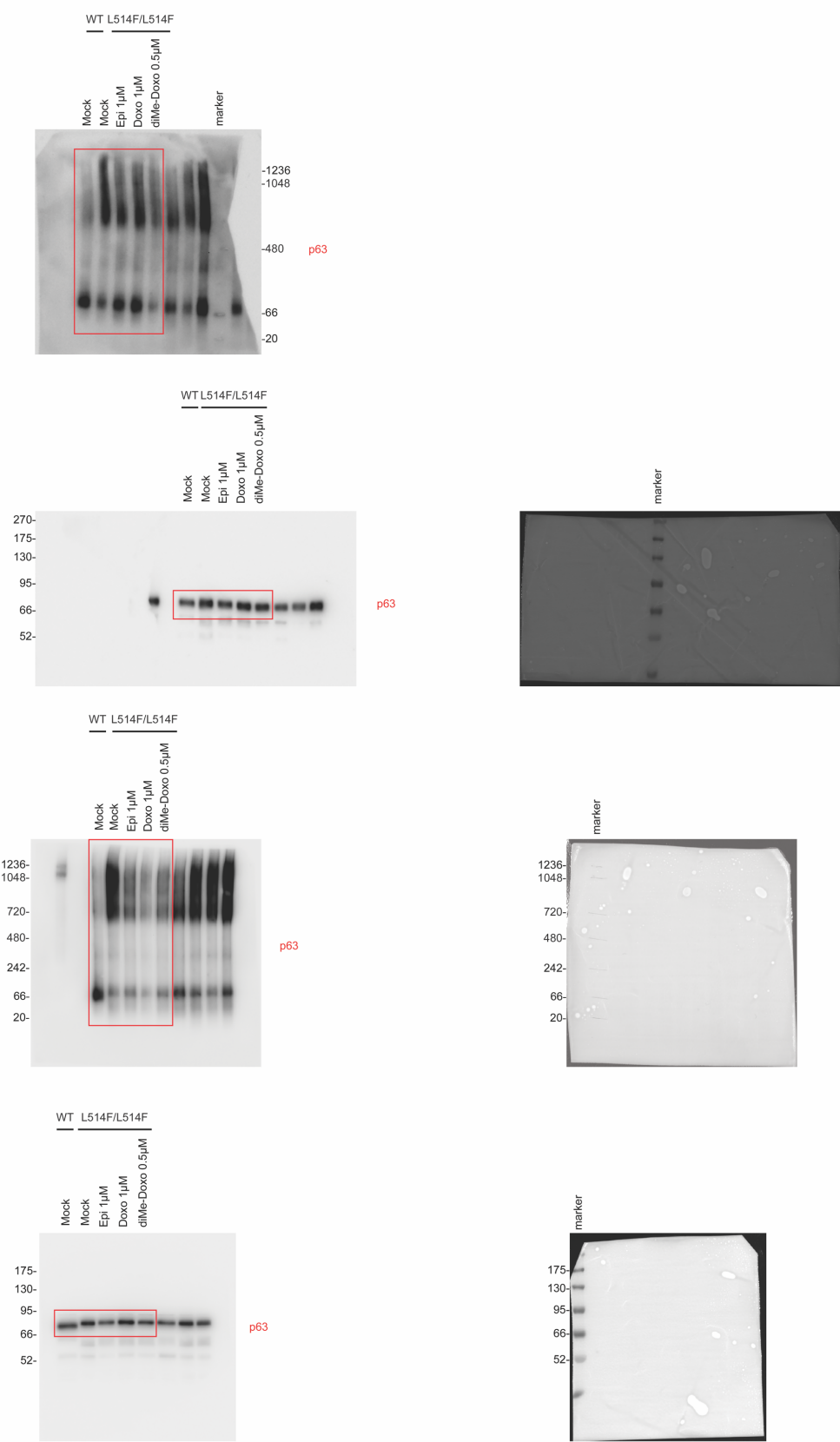

Figure S1

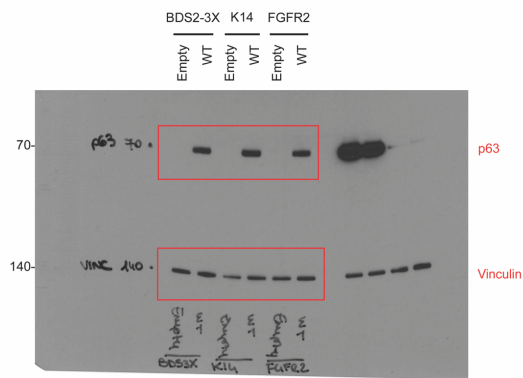

Figure S4

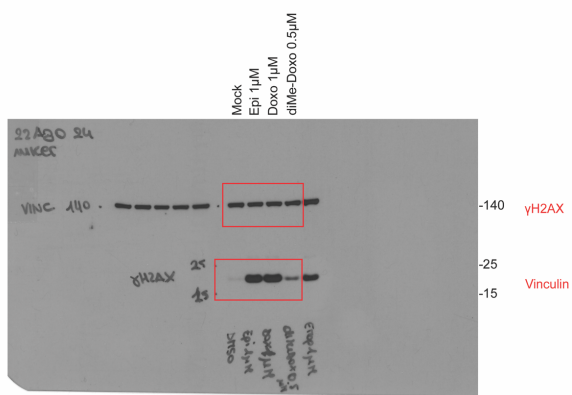

Supplement: Supplementary file 1 — Original DATA Western Blots [file 41420_2025_2307_MOESM1_ESM.pdf]
