# Supplementary material for: Anthracyclines disaggregate and restore mutant p63 function: a potential therapeutic approach for AEC syndrome
Source: Cell Death Discov. 2025 Jan 25;11:24. doi: 10.1038/s41420-025-02307-0 (PMC11762975; doi:10.1038/s41420-025-02307-0)
Supplement: Supplementary file 2 — Supplementary Information [file 41420_2025_2307_MOESM2_ESM.pdf]

Supplementary figure and figure legends

Figure S1

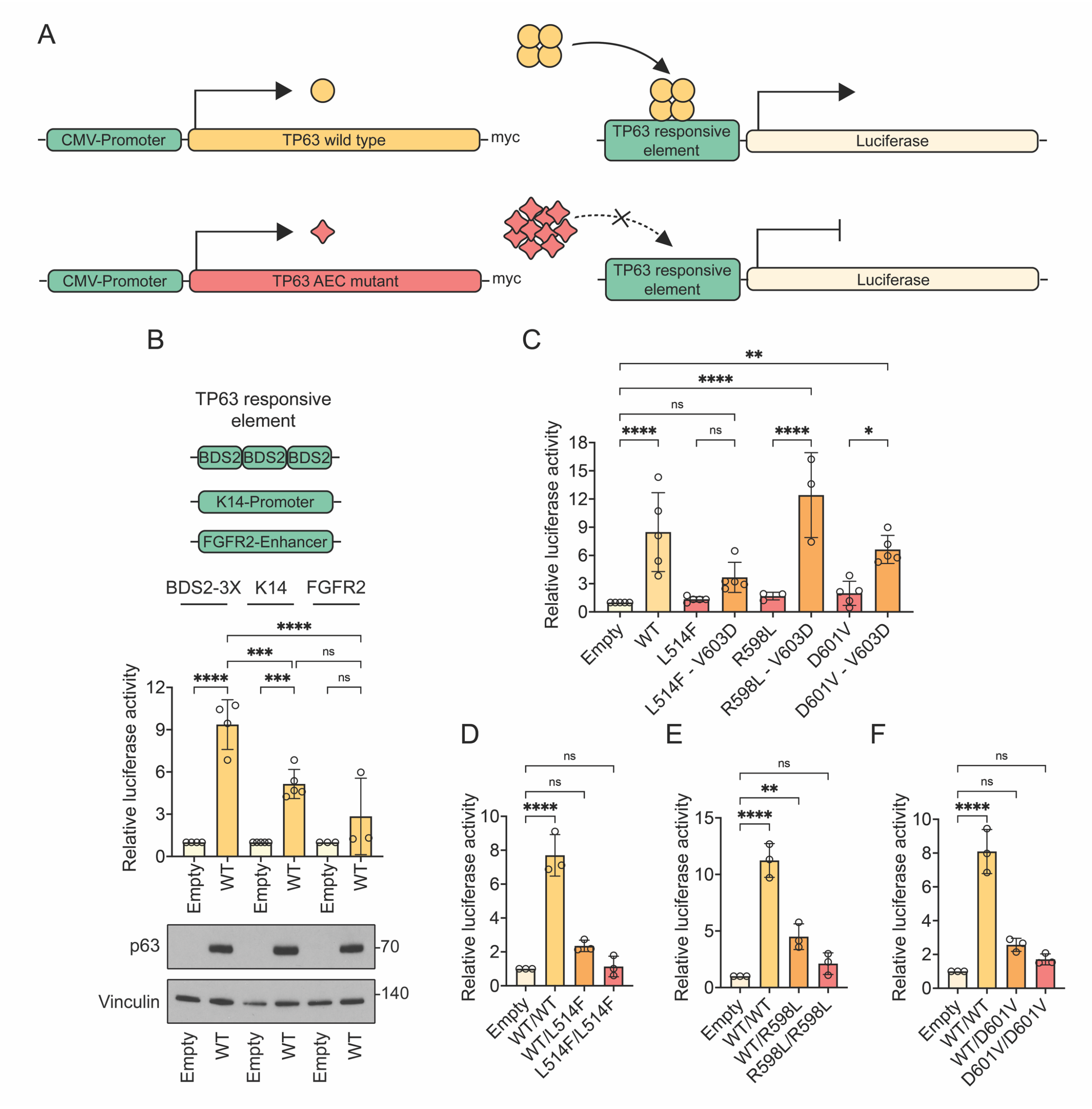

Figure S1. Establishment of HTS strategy. (A) Schematic representations of p63 constructs expressing for wild-type p63 or AEC mutants which, due to aggregation, are causative of the impairment of p63 transcriptional function. Plasmids were transfected into H1299 cells that lack of p63 and its family members. (B) Schematic representation of expression constructs carrying the luciferase reporter gene under different p63 responsive element (upper) and activity of the different promoters in

the luciferase reporter assay in H1299 cells transfected with wild-type p63 or empty vector (lower). Luciferase activity was measured 24 h after transfection. Relative luciferase activity was normalized to transfection with empty vector. SDS-PAGE followed by Western blot for p63 is shown as expression control. Vinculin was used as a loading control. (C) Luciferase reporter assays in H1299 transfected with empty vector, wild-type p63 or the indicated mutant p63 using the pBSD2 3X-luc. Relative luciferase activity was normalized to transfection with empty vector. (D-F) Luciferase reporter assay was performed in H1299 cells after 24 h transfection with empty vector, wild-type p63 alone or together with the mutant L514F, R598L and D601V, respectively. Relative luciferase activity was normalized to transfection with empty vector. All data are represented as mean ± SD ($n > 3$), *$p < 0.05$, **$p < 0.01$, ***$p < 0.001$, ****$p < 0.0001$ one way ANOVA test.

Figure S2

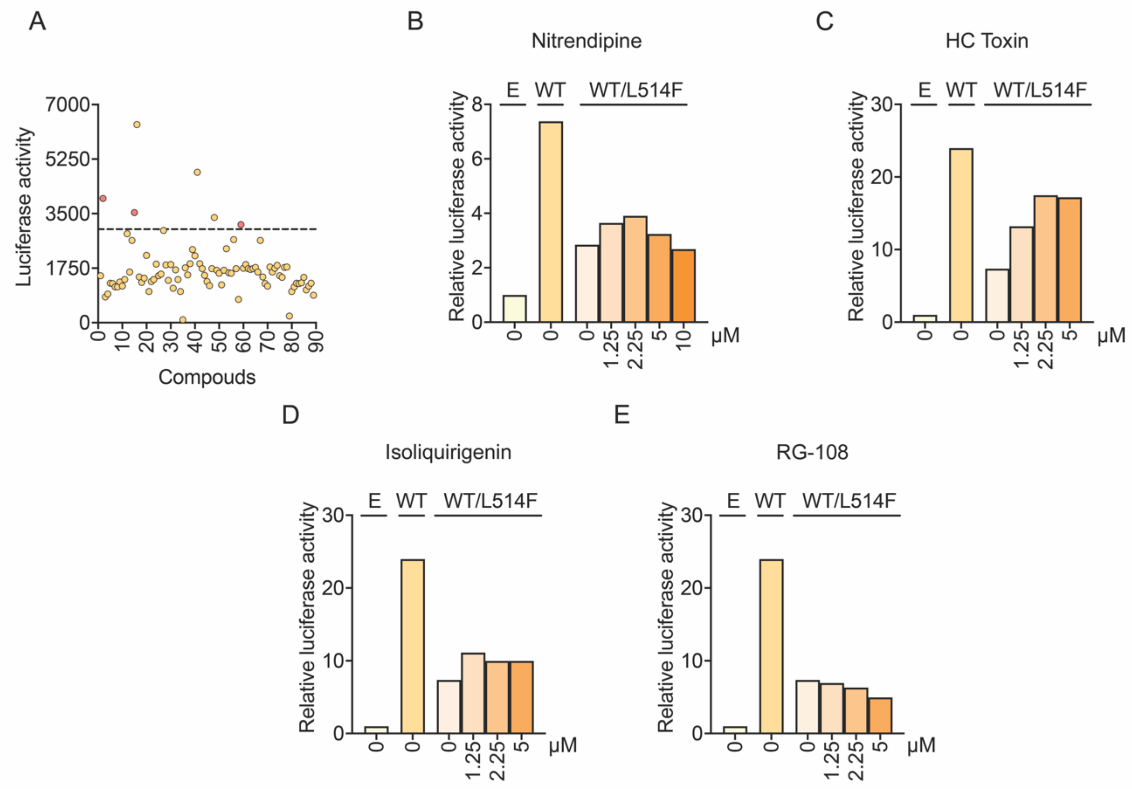

Figure S2. Validation of HTS hits. (A) Scatter plot of HTS results. A total of 88 epigenetics compounds were screened. Arbitrary cut-off threshold was set at a level 3000 of luciferase activity. Data points represent the average value of luciferase activity for each compound. The plate was run in triplicate. Statistically significant compounds are indicated in red. (B-E) The dose-responses of significant compounds selected by screening were examined by luciferase reporter assay in H1299 co-transfected with a combination of wild-type p63 and mutant L514F, wild-type p63 alone or empty vector. 24 h after transfection, cells were incubated at the indicated concentrations of the listed compounds for 24 h followed by analysis of luciferase activity. Relative luciferase activity was normalized to transfection with empty vector.

Figure S3

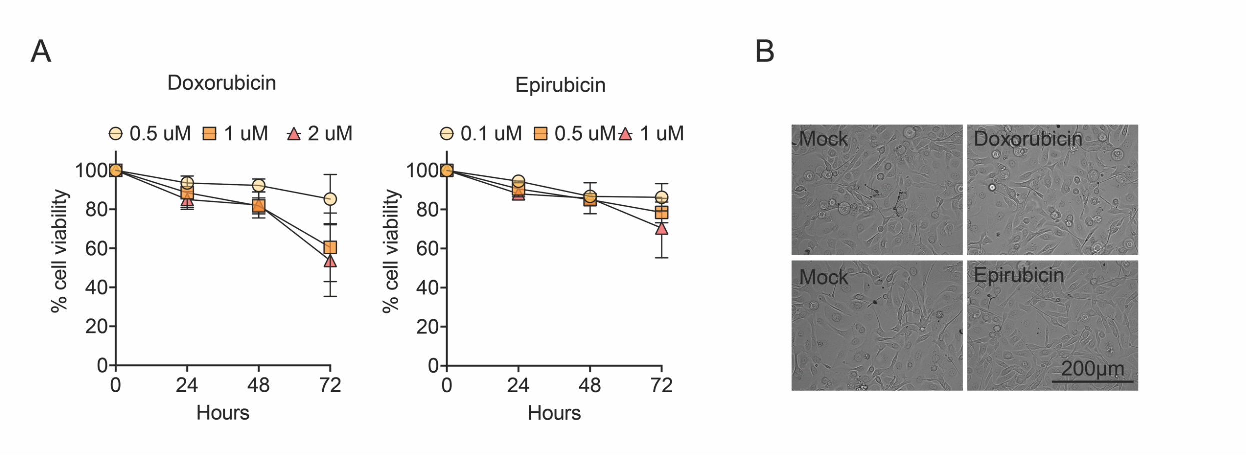

Figure S3. Doxo and Epi effects on cell viability of mouse primary keratinocytes. (A) MTT assays were performed in mouse primary keratinocytes after treatment with the indicated concentration of Doxo and Epi for 24, 48 and 72 h. Data are represented as mean ± SD (n = 4). (B) Representative bright field images of mouse primary keratinocytes after the treatment with 1 μM Doxo and 1 μM Epi for 24 h. Scale bar 200 μm.

Figure S4

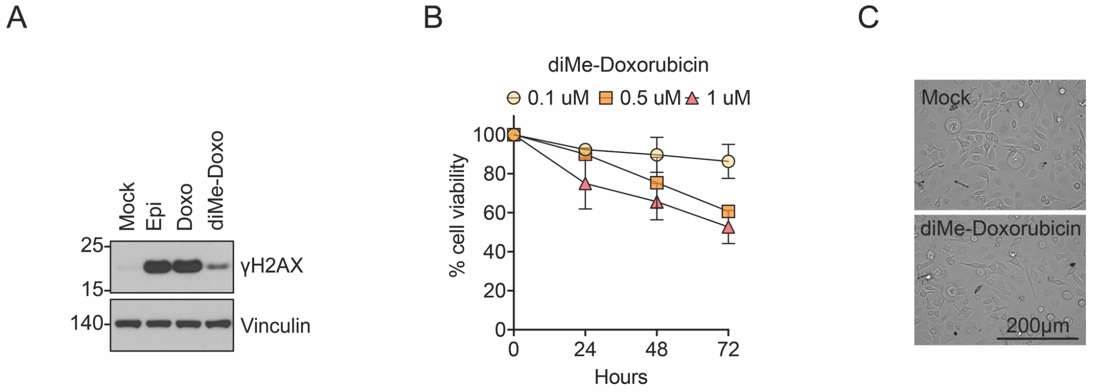

Figure S4. Effects of diMe-Doxo on cell survival. (A) SDS-PAGE followed by Western blot for γH2AX in mouse primary keratinocytes upon treatment with 1 μM Epi, 1 μM Doxo or 0.5 μM diMe-Doxo for 24 h. Vinculin was used as a loading control. (B) MTT assay was performed after treatment with the indicated concentration of diMe-Doxo for 24, 48 and 72 h. Data are represented as mean ± SD (n = 3). (C) Representative bright field images of mouse primary keratinocytes after the treatment with 0.5 μM diMe-Doxo for 24 h. Scale bar 200 μm.
